# Identification of a novel HLA-A24-restricted cytotoxic T lymphocyte epitope peptide derived from mesothelin in pancreatic cancer

**DOI:** 10.18632/oncotarget.25837

**Published:** 2018-07-31

**Authors:** Mariko Tsukagoshi, Satoshi Wada, Seiko Hirono, Shintaro Yoshida, Erica Yada, Tetsuro Sasada, Ken Shirabe, Hiroyuki Kuwano, Hiroki Yamaue

**Affiliations:** ^1^ Division of Hepatobiliary and Pancreatic Surgery, Department of General Surgical Science, Gunma University Graduate School of Medicine, Maebashi Gunma 371-8511, Japan; ^2^ Department of Innovative Cancer Immunotherapy, Gunma University Graduate School of Medicine, Maebashi Gunma 371-8511, Japan; ^3^ Department of General Surgical Science, Gunma University Graduate School of Medicine, Maebashi Gunma 371-8511, Japan; ^4^ Department of Cancer Immunotherapy, Kanagawa Cancer Center, Asahi-ku, Yokohama Kanagawa 241-8515, Japan; ^5^ Second Department of Surgery, Wakayama Medical University, Wakayama 641-8510, Japan

**Keywords:** mesothelin, epitope peptide, cancer vaccine, immunotherapy, pancreatic cancer

## Abstract

Pancreatic cancer involves highly malignant tumors, and the development of new therapeutic strategies is critical. Mesothelin is overexpressed in infiltrating pancreatic cancer cells and plays an important role in the invasion and migration processes. In this study, we focused on mesothelin as a tumor-specific antigen target for a pancreatic cancer vaccine. We first investigated the mesothelin-derived epitope peptide restricted to HLA-A^*^2402. A total of 19 candidate peptides were synthesized, and we then determined their potential to induce peptide-specific cytotoxic T lymphocytes (CTLs). Peptide-specific CTLs were induced by five peptides derived from mesothelin, and these CTLs successfully exhibited peptide-specific IFN-γ production. After the expansion of each CTL, two CTL lines were established, which were induced by mesothelin-10-5 peptide (AFYPGYLCSL). These CTL lines exhibited peptide-specific cytotoxicity and IFN-γ production. Moreover, we were able to generate mesothelin-10-5 peptide-specific CTL clones. These CTL clones also had specific cytotoxic activity against HLA-A^*^2402-positive pancreatic cancer cells that endogenously expressed mesothelin. These results indicate that the mesothelin-10-5 peptide is a novel HLA-A^*^2402 restricted CTL epitope and that it is a promising candidate target for antigen-specific immunotherapy against pancreatic cancers.

## INTRODUCTION

Pancreatic cancer is one of the most lethal malignancies and is associated with poor prognosis, and the 5-year relative survival rate is currently 8% [1]. This low rate is partly due to the fact that more than one-half of cases are diagnosed at a late stage, and therapeutic modalities are very limited [1, 2]. Although surgical resection is the only curative treatment for pancreatic cancer, even the 5-year survival rate after a curative resection is only ∼20% [3]. Therefore, the identification of novel therapeutic modalities is critical for improving patient prognosis.

Recently, the development of cancer immunotherapy has considerably expanded. In particular, immune checkpoint inhibitors such as anti-cytotoxic T-lymphocyte antigen 4 (CTLA-4), anti-programmed death 1 (PD-1), and anti-programmed death ligand 1 (PD-L1) antibodies represent significant recent developments. Although immune checkpoint inhibitors have successfully achieved durable antitumor responses in a variety of malignant diseases, only a fraction of patients respond to treatment, and objective response rates have ranged from approximately 10% to 30% in clinical trials [4, 5]. Moreover, immune checkpoint inhibitors have been shown to be ineffective as single agents in the treatment of pancreatic cancer and to not prolong survival [6, 7]. Hence, combined therapeutic approaches are necessary to achieve complete tumor regression and are being evaluated [8]. A recent study reported that immune checkpoint inhibitors combined with a cancer vaccine may alter the microenvironment from a cold tumor to a hot tumor and enhance the vaccine-induced anti-tumor response [9]. Therefore, we explored potential targets for combination therapy with immune checkpoint inhibitors in pancreatic cancer.

Pancreatic cancer is aggressive and characterized by invasiveness, rapid progression, and profound resistance to treatment [10]. The invasion and metastasis processes are crucial to the pathogenesis of pancreatic cancer; however, their genetic and biochemical determinants are still not fully understood [11]. We previously identified specific genes, MUC16 and mesothelin, associated with invasion and migration processes in pancreatic cancer [12]. We reported that MUC16 and mesothelin were overexpressed only in infiltrating pancreatic cancer cells but not in PanIN-3 cells or normal pancreatic tissues. It has also been reported that mesothelin may play an important role in cell adhesion [13], cell proliferation and migration, tumor progression [14, 15], and resistance to chemotherapy [16]. Thus, mesothelin might be an attractive target for the development of novel cancer treatments for pancreatic cancer.

In the present study, we focused on mesothelin as a novel tumor-specific antigen target for anticancer immunotherapy for pancreatic cancer. We investigated the mesothelin-derived epitope peptide restricted to HLA-A^*^2402 that can induce peptide-specific cytotoxic T lymphocytes (CTLs) for possible use in peptide-based immunotherapies for patients with HLA-A^*^ 2402-positive pancreatic cancer.

## RESULTS

### CTL responses to candidate peptides derived from mesothelin

**Figure 1:**
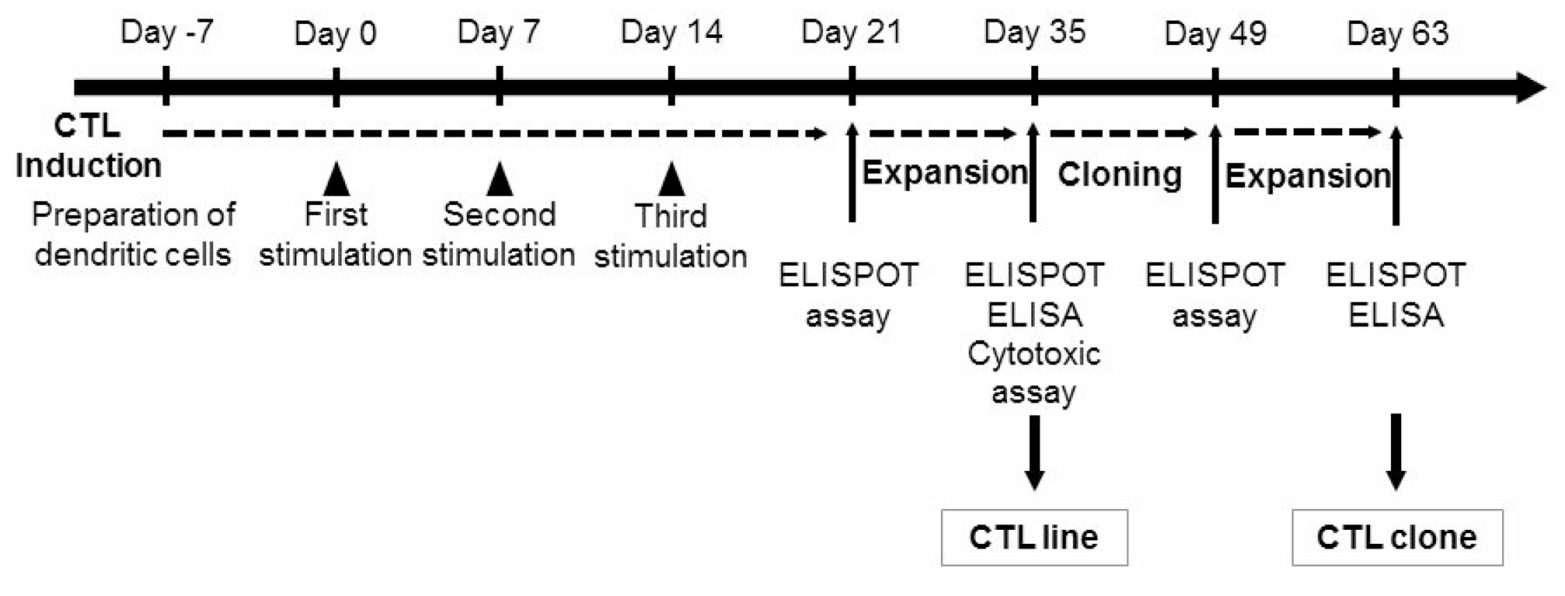
The protocol used for the identification of peptide-specific CTLs CTLs were induced by a three-time stimulation with DCs loaded with mesothelin-derived peptides. Then, CTLs were examined for specificity for each peptide using IFN-γ ELISPOT assays. Peptide-specific CTLs obtained from ELISPOT-positive wells were expanded and then examined with an IFN-γ ELISPOT assay and ELISA and cytotoxic assay. Peptide-specific CTL clones were established using the limiting dilution method and examined using an IFN-γ ELISPOT assay and ELISA.

**Figure 2:**
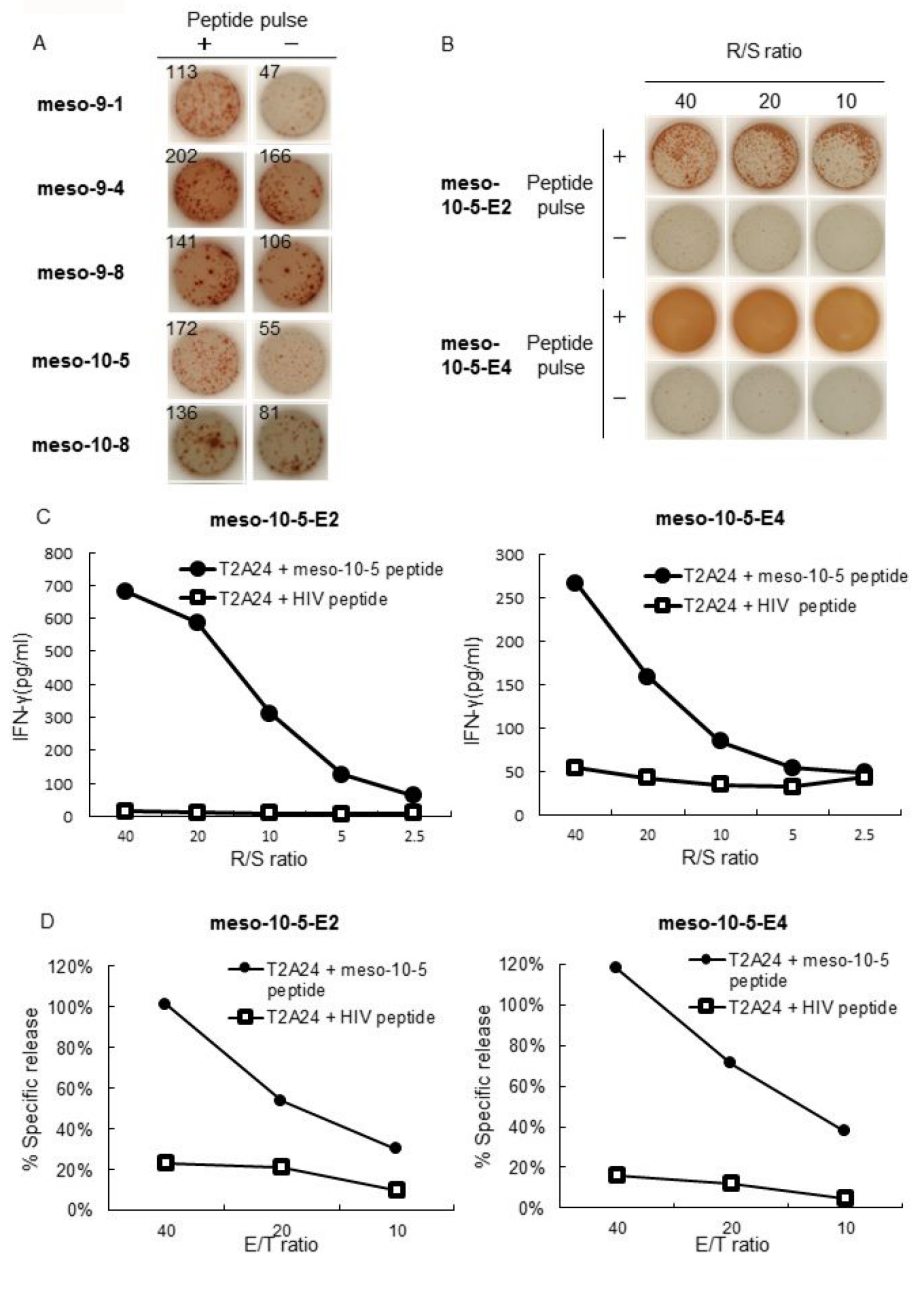
IFN-γ production and cytotoxic activity of peptide-specific CTLs (**A**) CTLs induced by mesothelin-derived peptides were stimulated with T2A24 cells pulsed with or without mesothelin-derived peptides. IFN-γ production by CTLs in response to the meso-9-1, meso-9-4, meso-9-8, meso-10-5, or meso-10-8 peptides was examined using an IFN-γ ELISPOT assay. The numbers indicate the IFN-γ ELISPOT counts per well. (**B**) IFN-γ production by CTL lines induced with meso-10-5 peptide stimulation after CTL-expanding culture was examined with an IFN-γ ELISPOT assay. R/S ratio, responder/stimulator ratio. (**C**) Meso-10-5 peptide-specific CTL line activity was examined with an IFN-γ ELISA. R/S ratio, responder/stimulator ratio. (**D**) The cytotoxic activity of the mesothelin-10-5 peptide-specific CTL lines was examined with a fluorescence-based cytotoxicity assay. E/T ratio, effector/target ratio.

To identify mesothelin-derived and HLA-A24-restricted CTL epitopes, we synthesized a total of 19 candidate 9-mer and 10-mer peptides that were expected to have high binding affinity to HLA-A24 using the BIMAS program (Table 1). These peptides were evaluated for their potential to induce peptide-specific CTLs *in vitro* from human PBMCs obtained from HLA-A24 healthy donors (Figure 1). CTLs were induced by a three-time stimulation with DCs loaded with mesothelin-derived peptides. Then, CTLs were examined for specificity for each peptide using IFN-γ ELISPOT assays. Peptide-specific CTLs were judged to have been successfully induced when there were more than 100 spot counts for each and 30 more spot counts than the control. Five peptides could induce peptide-specific CTLs that produced IFN-γ and were able to specifically recognize T2-A24 cells pulsed with each peptide but not T2-A24 cells not pulsed with peptides (Figure 2A).

**Table 1:** Candidate peptides derived from mesothelin restricted with HLA-A^*^24:02

Peptide name	Position	Amino acid sequence (mer)	Binding score
meso-9-1	443–451	FYPGYLCSL (9)	300
meso-9-2	483–491	LYPKARLAF (9)	150
meso-9-3	498–506	EYFVKIQSF (9)	120
meso-9-4	424–432	RFVKGRGQL (9)	60
meso-9-5	592–600	GYLVLDLSM (9)	45
meso-9-6	499–507	YFVKIQSFL (9)	42
meso-9-7	413–421	RPLPQVATL (9)	14.4
meso-9-8	306–314	KAREIDESL (9)	13.4
meso-9-9	206–214	RLVSCPGPL (9)	12
meso-9-10	429–437	RGQLDKDTL (9)	12
meso-10-1	362–371	GYPESVIQHL (10)	604.8
meso-10-2	498–507	EYFVKIQSFL (10)	280
meso-10-3	194–203	RFVAESAEVL (10)	60
meso-10-4	67–76	GFPCAEVSGL (10)	30
meso-10-5	442–451	AFYPGYLCSL (10)	24
meso-10-6	182–191	RALGGLACDL (10)	14.4
meso-10-7	91–100	KNVKLSTEQL (10)	12
meso-10-8	385–394	KWNVTSLETL (10)	12
meso-10-9	548–557	KLLGPHVEGL (10)	12

The binding score was obtained from the BIMAS program (https://www-bimas.cit.nih.gov/molbio/hla_bind/).

### Establishment of CTL lines that respond to epitope peptides derived from mesothelin

After CTL-expanding culture, only the mesothelin-10-5 peptide-specific CTLs produced a large amount of IFN-γ in ELISPOT assays (Figure 2B). CTL activity was also examined by IFN-γ ELISA. The mesothelin-10-5 peptide-specific CTLs produced a large amount of IFN-γ specifically in response to T2A24 cells pulsed with the mesothelin-10-5 peptide but not to T2A24 cells with HIV peptide loading (Figure 2C). Similarly, in independent experiments using PBMCs from two other healthy donors, mesothelin-10-5 peptide could induce CTLs that produced high levels of IFN-γ (data not shown).

The cytotoxic activity of the mesothelin-10-5 peptide-specific CTLs was examined using a fluorescence-based cytotoxicity assay. The mesothelin-10-5 peptide-specific CTLs demonstrated cytotoxic activity against T2A24 cells pulsed with the mesothelin-10-5 peptide but not against T2A24 cells with HIV peptide loading (Figure 2D).

### Establishment of mesothelin-derived peptide-specific CTL clones

**Figure 3:**
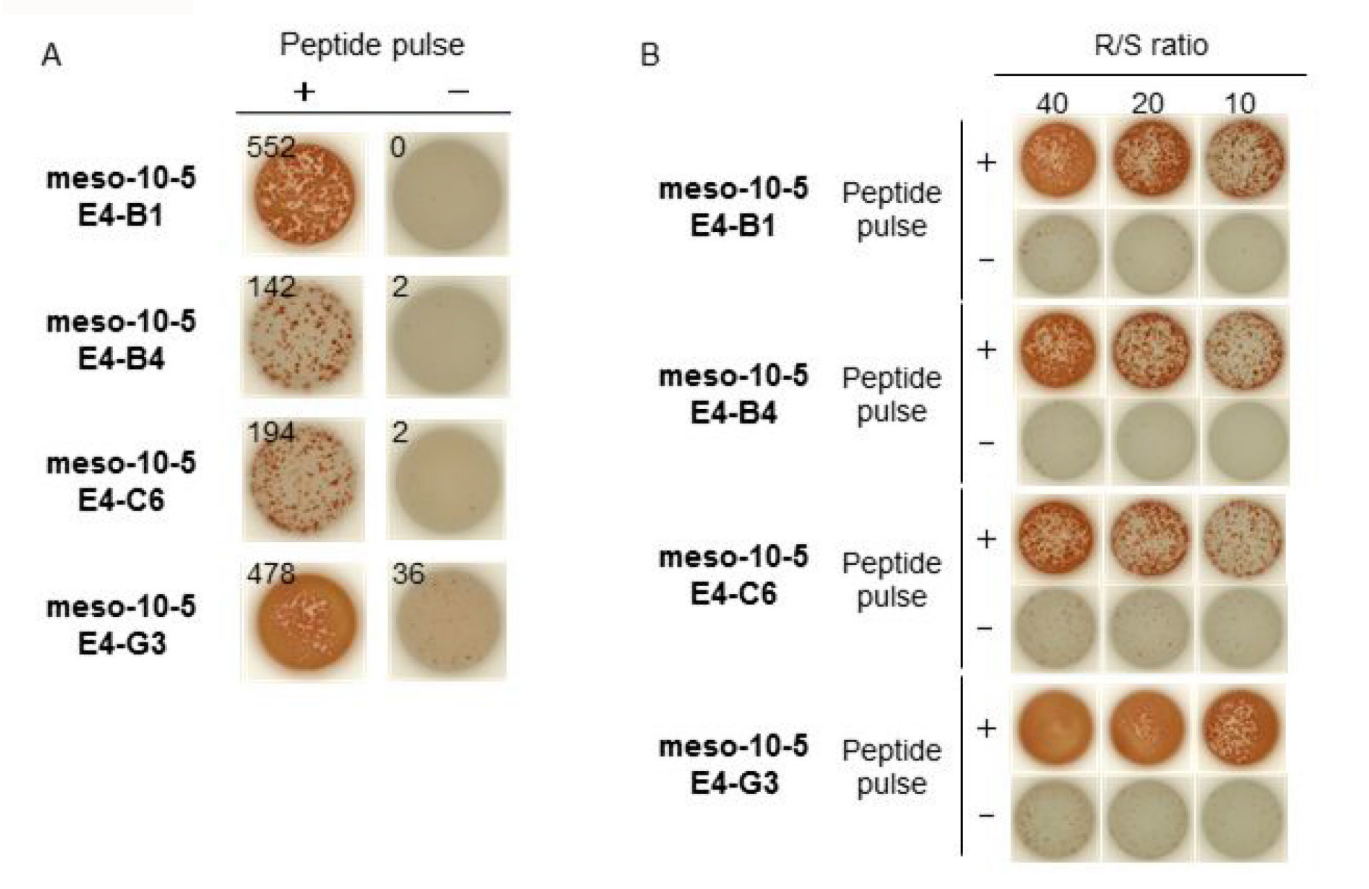
IFN-γ production by meso-10-5 peptide-specific CTL clones (**A**) Four established mesothelin-10-5 peptide-specific CTL clones were stimulated with T2A24 cells pulsed with or without meso-10-5 peptides. IFN-γ production by CTL clones was examined using an IFN-γ ELISPOT assay. The numbers indicate the IFN-γ ELISPOT counts per well. (**B**) IFN-γ production by CTL clones induced with meso-10-5 peptide stimulation after CTL clone expansion was examined with an IFN-γ ELISPOT assay. R/S ratio, responder/stimulator ratio.

We next extracted CTL clones from the CTL lines and finally established four CTL clones using a limiting dilution method using CTL lines that responded to mesothelin-10-5 peptide (Figure 3A). The established mesothelin-10-5 peptide-specific CTL clones produced a high level of IFN-γ specifically in response to mesothelin-10-5-pulsed T2A24 cells, whereas no IFN-γ production was detected against T2A24 cells not pulsed with the peptide, even after CTL clone expansion (Figure 3B).

### Peptide-specific CTL response to mesothelin and HLA-A^*^2402-expressing pancreatic cancer cells

**Figure 4:**
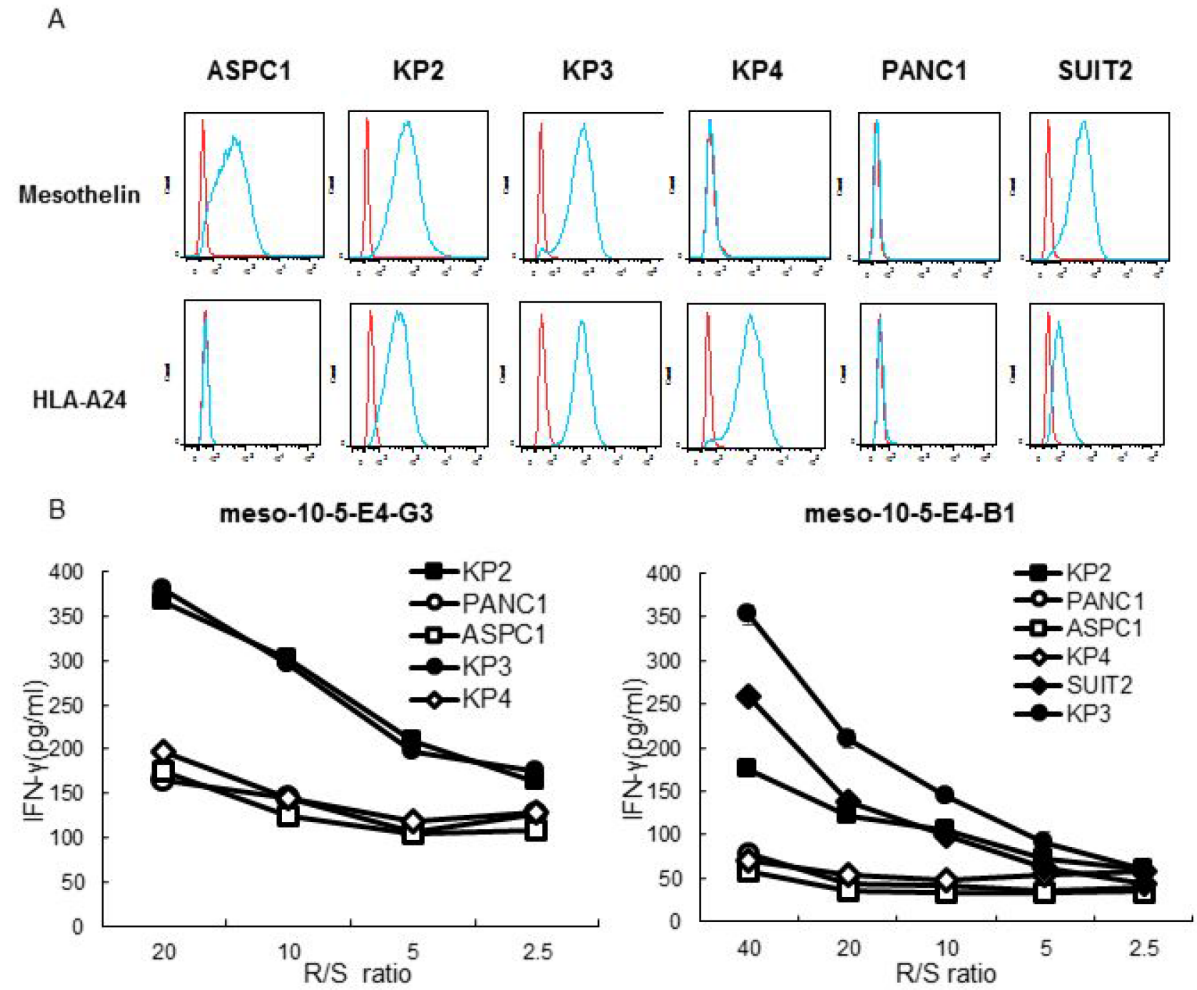
Peptide-specific CTL response to mesothelin and HLA-A^*^2402-expressing pancreatic cancer cells (**A**) Flow cytometry analysis of the surface expression of mesothelin and HLA-A24 on human pancreatic cancer cell lines: ASPC1, KP2, KP3, KP4, PANC1, and SUIT2. Red line, isotype control. Blue line, mesothelin or HLA-A24 staining. Top row: using APC mouse anti-human isotype antibody and mouse anti-human mesothelin antibody. Bottom row: using PE mouse anti-human isotype antibody and PE mouse anti-human HLA-A24 antibody. (**B**) IFN-γ production by mesothelin-10-5 peptide-specific CTL clones against pancreatic cancer cell lines was examined with an IFN-γ ELISA. R/S ratio, responder/stimulator ratio.

The responses of the established mesothelin-10-5 peptide-specific CTL clones were examined. The pancreatic cancer cell lines KP2, KP3, ASPC1, and SUIT2 expressed mesothelin, whereas PANC1 and KP4 did not. Furthermore, the KP2, KP3, KP4, and SUIT2 cell lines expressed HLA-A^*^2402, whereas ASPC1 and PANC1 cells did not (Table 2 and Figure 4A). The mesothelin-10-5 peptide-specific CTL clones produced more IFN-γ against HLA-A^*^2402-positive and mesothelin-expressing cell lines (KP2, KP3, and SUIT2 cells) than cell lines lacking either HLA-A^*^2402 or mesothelin (ASPC1, PANC1, and KP4 cells) (Figure 4B).

**Table 2:** Antigen expression in pancreatic cancer cell lines

Pancreatic cancer cell lines	Antigen expression	
	mesothelin	HLA-A 2402
ASPC1	+	−
KP2	+	+
KP3	+	+
KP4	−	+
PANC1	−	−
SUIT2	+	+

### Assessment of therapeutic efficacy of peptide-specific CTLs *in vivo*

**Figure 5:**
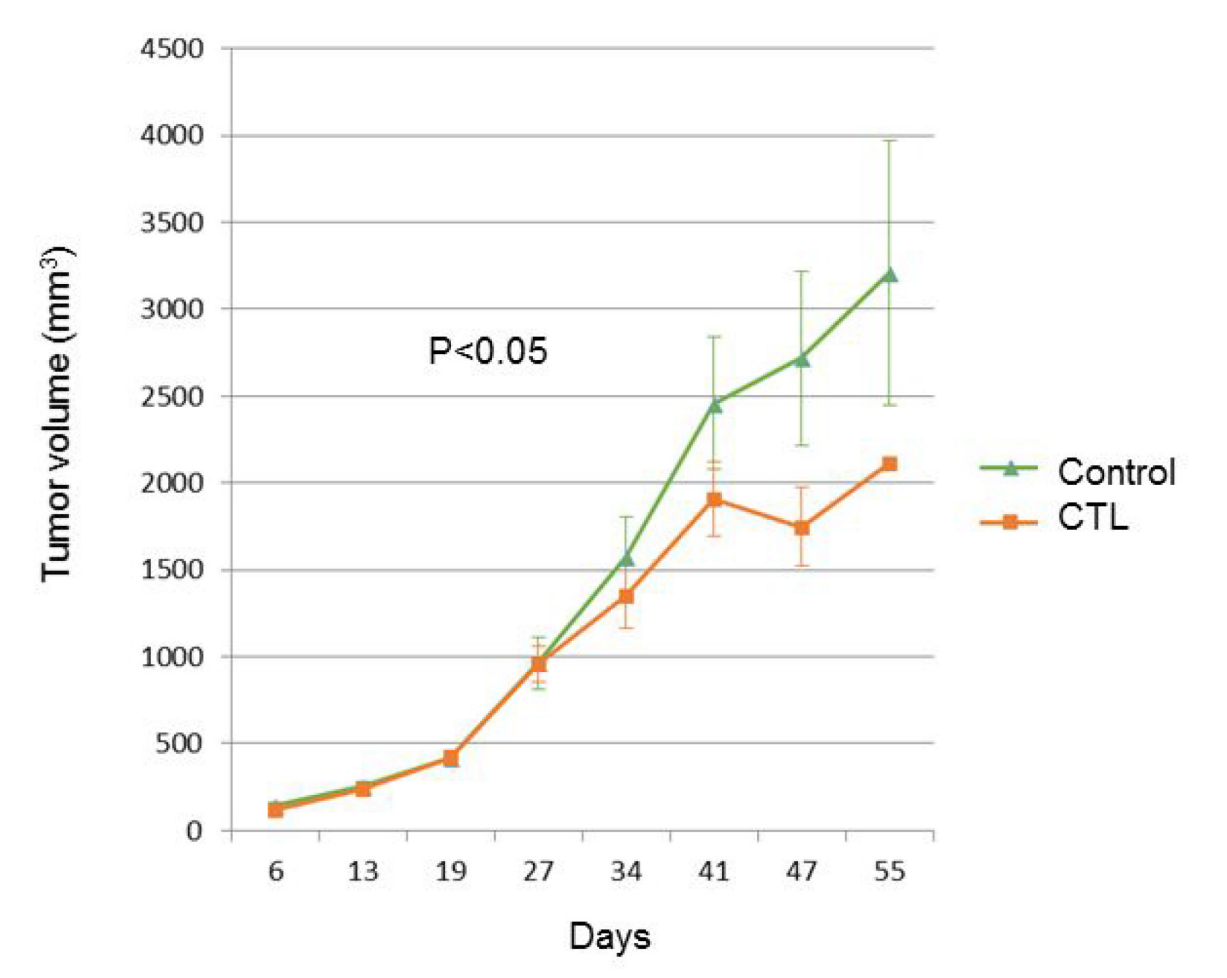
The tumor growth curve of SUIT2-xenograft tumors with or without mesothelin-specific CTL injection

We investigated whether peptide-specific CTL treatment promotes pancreatic cancer rejection in mice. Treatment with peptide-specific CTL showed SUIT2 tumor regression in NSG mice, whereas a progressive increase in tumor growth was observed in control groups (*p* < 0.05) (Figure 5).

## DISCUSSION

In the current study, we identified a new mesothelin-derived epitope peptide restricted to HLA-A^*^2402. We successfully induced peptide-specific CTL lines that exhibited peptide-specific IFN-γ production and cytotoxicity against T2A24 cells pulsed with the mesothelin-10-5 peptide (AFYPGYLCSL). Furthermore, we were able to establish mesothelin peptide-specific CTL clones. The peptide-specific CTL clones also exhibited peptide-specific IFN-γ production against HLA-A^*^2402-positive pancreatic cancer cells that endogenously expressed mesothelin. These results suggest that targeting mesothelin could be a novel approach for developing cancer vaccines for pancreatic cancer.

Mesothelin has been shown to be overexpressed in many human cancers, including mesothelioma, ovarian cancer, pancreatic cancer, lung cancer, gastric cancer, and biliary cancer [12, 17–22]. Furthermore, high expression of mesothelin was correlated with poor prognosis in several human cancers [22–24], and in pancreatic cancer, the coexpression of MUC16 and mesothelin was reported to be an independent prognostic factor for poor prognosis [12]. However, the expression and biological functions of mesothelin in cancer progression remain poorly understood. Recent studies have revealed that mesothelin may play an important role in cancer survival/proliferation, tumor progression, and drug resistance through the Wnt/NF-kB/PI3K/Akt signaling pathway [14, 16, 25]. Furthermore, recent studies have revealed that mesothelin is associated with the invasion and migration of pancreatic cancer cells [12, 26]. Thus, mesothelin might be an attractive therapeutic target for pancreatic cancer, and we focused on this protein for developing a cancer vaccine using an epitope peptide.

Several immunotherapy agents targeting mesothelin have been developed, and some trials have shown that targeting mesothelin is safe and does not result in toxicity to essential normal tissues [27, 28]. Mesothelin tumor vaccines are currently being evaluated, and their safety was established in a phase I clinical trial of patients with mesothelin-expressing advanced cancers, including pancreatic cancer [29]. In this study, we identified an HLA-A^*^2402-restricted novel epitope peptide derived from mesothelin. Our established epitope peptide-specific CTL clones responded to pancreatic cancer cells that endogenously expressed mesothelin in an HLA-A24-restricted manner. These results suggest that the mesothelin-10-5 peptide (AFYPGYLCSL) is naturally processed from mesothelin in pancreatic cancer cells and presented on the cell surface with the HLA-A24 molecule. Thus, mesothelin-10-5 peptide-specific CTLs might exert an antitumor effect against mesothelin-expressing pancreatic cancer cells in HLA-A24-positive patients.

Although cancer vaccines can induce an effective anti-tumor T cell response, a vaccine alone may be insufficient to induce a complete tumor cell-killing effect. An optimal anti-tumor immune response requires not only an increase in immune activation but also a decrease in immune suppression. The upregulation of PD-L1 has been reported in a wide variety of cancers, including pancreatic cancer, and the PD-1/PD-L1 pathway suppresses the anti-tumor immune response [30]. PD-L1 expression on a tumor can be induced thorough oncogenic pathways [31] or an adaptive immune resistance mechanism in response to the production of inflammatory cytokines by tumor-infiltrating lymphocytes [32–34]. Recent studies have reported that tumor-specific T cells were recruited to the tumor microenvironment and that immune checkpoint expression, including PD-L1, was upregulated in response to therapeutic vaccines in pancreatic cancer [9, 35]. Thus, the tumor microenvironment could be changed from a cold tumor to a hot tumor by a cancer vaccine. To overcome this cold tumor problem in which an anti-tumor effect is not achieved with a checkpoint inhibitor alone, a cancer vaccine to induce CTLs combined with checkpoint blockade antibodies that inhibit immune suppression represents a good combination therapy to mediate complete tumor suppression [36].

In addition to using vaccine-based therapies, other approaches such as T cell transfer therapies are also under consideration for immunotherapy. Chimeric antigen receptor T cells (CAR-T cells) are produced by one method for adoptive T cell transfer, and a preliminary study showed that mesothelin-specific CAR mRNA-engineered T cells could induce an anti-tumor immune response [37]. A recent study has shown that checkpoint inhibitors could also enhance the CAR-T cell response [38]. These data suggest that mesothelin is a good target for cancer immunotherapy. Peptide vaccines have several advantages, including that peptide synthesis can be performed easily at a low cost and that their relative safety has been demonstrated in preclinical and clinical studies. Therefore, peptide cancer vaccines may be a good option for use in combination therapies.

In conclusion, we identified a novel HLA-A24-restricted epitope peptide derived from mesothelin. Mesothelin is a promising target for peptide-based immunotherapy for patients with pancreatic cancer. Furthermore, a mesothelin peptide vaccine might be useful in future combination therapies with checkpoint inhibitors. Further investigations of the safety and efficacy of a mesothelin peptide vaccine are needed before clinical application.

## MATERIALS AND METHODS

### Peptides

Mesothelin-derived 9-mer and 10-mer peptides with high binding affinity to HLA-A:^*^24:02 were predicted with the binding prediction software ‘‘BIMAS’’ (http://www-bimas.cit.nih.gov/molbio/hla_bind). HLA-A^*^24:02-restricted HIV-derived epitope peptide (RYLKDQQLL) was used for a negative control, and HLA-A24 restricted CMV peptide (QYDPVAALF) was used for a positive control. Peptides were dissolved in dimethylsulfoxide at 20 mg/ml and stored at −80° C.

### Cell lines

T2A24 (HLA-A^*^02:01, HLA-A^*^24:02, lymphoblast) cells were provided by Dr. Kuzushima (Aichi Cancer Center) [39]. Jiyoye (HLA-A32, B17, Bw37, Burkitt’s lymphoma), EB-3 (HLA-A3, Aw32, Cw2, Burkitt’s lymphoma), PANC-1 (pancreatic cancer), and ASPC1 (pancreatic cancer) cells were purchased from the American Type Culture Collection (Manassas, VA). T2A24 cells were maintained in RPMI1640 media with HEPES (Thermo Fisher Scientific, Waltham, MA) supplemented with 10% heat-inactivated fetal bovine serum (FBS; Biowest, France) and 0.8 mg/ml Geneticin^®^ Selective Antibiotic (Thermo Fisher Scientific, Waltham, MA). Jiyoye and EB-3 cells were maintained in RPMI1640 media (Thermo Fisher Scientific, Waltham, MA) supplemented with 10% heat-inactivated FBS and 1% penicillin-streptomycin (liquid, Thermo Fisher Scientific, Waltham, MA). KP2, KP3, KP4, and SUIT2 cells were purchased from the *JCRB* Cell Bank (Japan). The expression levels of HLA-A^*^2402 and mesothelin were examined by flow cytometry with an anti-HLA-A^*^2402 monoclonal antibody and anti-mesothelin antibody to select HLA-A^*^2402- and mesothelin-positive target cancer cells.

### *In vitro* induction of peptide-specific CTLs

Peptide-specific CTLs were induced as described previously [40, 41]. CD8^+^ T cells and dendritic cells (DCs) were prepared from peripheral blood mononuclear cells (PBMCs) obtained from HLA-A^*^24:02-positive healthy volunteers after receiving their written informed consent. This study was approval by an appropriate ethics committee, and all clinical procedures involving human subjects followed the principles expressed in the Declaration of Helsinki. PBMCs were isolated with Ficoll-Paque PLUS (GE Healthcare, Uppsala, Sweden) and separated into CD8^+^ T cell and CD8^−^ cell populations with a human CD8+ T Cell Isolation Kit (Miltenyi Biotec K.K., Japan). The monocyte-enriched CD8^−^ cell population was cultured in AIM-V medium (Invitrogen) containing 2% heat-inactivated autologous serum (AS). After an overnight incubation, nonadherent cells were washed out, and 1,000 U/ml granulocyte-macrophage colony-stimulating factor (GM-CSF; R&D Systems, Minneapolis, MN) and 1,000 U/ml interleukin (IL)-4 (R&D Systems) were added to the culture to induce monocyte-derived DCs. On day 4, 0.1 KE/ml OK-432 (Chugai Pharmaceutical Co., Tokyo, Japan) was added to the culture to induce the maturation of DCs. On day 7, DCs were pulsed with 20 µg/ml the respective synthesized peptides in AIM-V medium containing 3 µg/ml β2- microglobulin (Sigma-Aldrich, ST. Louis, MO) at 37° C for 3 h.

These peptide-pulsed DCs were then incubated in media containing 30 µg/ml mitomycin C (MMC) (Kyowa Hakko Kirin Co. Ltd., Tokyo, Japan) at 37° C for 30 min. After washing out the residual peptides and MMC, DCs were cultured with autologous CD8^+^ T cells on 48 well plates (Corning, Inc., Corning, NY) (each well contained 1.5 × 10^4^ peptide-pulsed DCs, 3 × 10^5^ CD8^+^ T cells, and 10 ng/ml IL-7 (R&D Systems) in 0.5 ml of AIM-V/2% AS). Two days later, human IL-2 (Pepro Tech, Rocky Hill, NJ, USA) was added to the culture (final concentration: 20 IU/ml). On days 14 and 21, T cells were further re-stimulated with the autologous peptide-pulsed DCs, which were freshly prepared each time. On day 28, interferon-γ (IFN-γ) production was examined by IFN-γ enzyme-linked immunospot (ELISPOT) assay under stimulation with peptide-pulsed T2A24 cells.

### IFN-γ enzyme-linked immunospot (ELISPOT) assay

Peptide-specific CTL responses were investigated using an ELISPOT assay with an IFN-γ ELISPOT kit and AEC substrate set (BD Biosciences, San Jose, CA) according to the manufacturer’s instructions. For use as the stimulator cells, T2A24 cells were pulsed with 20 μg/ml the respective peptide at 37° C for 20 h, and residual peptide that did not bind to cells was washed out. After removing 500 μl of supernatant from each well of the induced peptide-specific CTLs, 100 μl of cell culture suspensions was harvested from each well and co-cultured with stimulator cells (1 × 10^4^ cells/well) on an ELISPOT plate at 37° C for 20 h. The resulting spots were counted using an ELIPHOTO Counter (Minerva Tech K.K., Japan).

### CTL expansion culture

Peptide-specific CTLs obtained from ELISPOT-positive wells after *in vitro* CTL induction were expanded as described previously [42, 43]. Suspensions of the remaining peptide-specific CTLs were co-cultured with 5 × 10^6^ MMC-treated (30 μg/ml at 37° C for 30 min) Jiyoye and EB-3 cells in 25 ml of 5% Auto serum/AIM-V containing 40 ng/ml anti-CD3 monoclonal antibody (BD Pharmingen, San Diego, CA) on day 0. IL-2 was added (final concentration: 120 IU/ml) on day 1, and 5% Auto serum/AIM-V containing 30 IU/ml IL-2 was provided on days 5, 8, and 11. On day 14, expanded CTL activity was examined using an IFN-γ ELISPOT assay.

### Establishment of peptide-specific CTL clones

Peptide-specific CTL clones were established by the limiting dilution method. CTLs were diluted to 0.3, 1, and 3 cells/well in 96-well round-bottom plates (Corning, Inc.) and co-cultured with 1 × 10^4^ cells/well of MMC-treated (30 μg/ml at 37° C for 30 min) Jiyoye and EB-3 cells in 150 µl of 5% Auto serum/AIM-V supplemented with 30 ng/ml anti-CD3 monoclonal antibody and 125IU/ml IL-2 on day 0. On day 10, IL-2 was added to each well (125 UI/ml). On day 14, IFN-γ an ELISPOT assay was performed to measure the CTL activity of each clone.

### Cytotoxicity assay

The cytotoxic activity of peptide-specific CTLs was examined using flow cytometry in a fluorescence-based cytotoxicity assay. In the flow cytometry analysis, both carboxyfluorescein diacetate succinimidyl ester (CFSE) and mesothelin peptide-pulsed cells were used as the target cells, and HIV peptide-pulsed and CFSE-labeled cells were used as controls. Target and control cells were labeled with 10 μM CFSE for 5 min each and co-incubated with CTL in 200 μl/well of 5% AS/AIM-V on a 96-well round-bottom plate for 4 h. Cell culture suspensions were harvested and analyzed using a FACS-Canto II flow cytometer (Becton Dickinson, San Jose, CA).

Fluorescence-based cytotoxicity assays were carried out with DELFIA EuTDA Cytotoxicity Assay Reagents (PerkinElmer Life and Analytical Sciences, Boston, MA). Mesothelin peptide-pulsed cells and HIV peptide-pulsed cells were used as the target cells and labeled with DELFIA BATDA reagent according to the manufacturer’s instructions for 30 min at 37° C. Then, the target cells with BATDA were incubated with peptide-specific CTLs for 4 h. After incubation, the supernatants were collected, and the time-resolved fluorescence was measured. Specific release was calculated as follows:

% Specific release = [(Experimental release – Spontaneous release)/(Maximum release – Spontaneous release)] × 100.

### IFN-γ enzyme-linked immunosorbent assay (ELISA)

The CTL activity was examined using an IFN-γ ELISA. Peptide-pulsed cells (1 × 10^4^ cells/well) or pancreatic cancer cells (5 × 10^4^ cells/well) were used as a stimulator. CTLs were co-cultured with stimulator cells in 200 μl/well of 5% AS/AIM-V on 96-well round bottom plates for 20 h. After incubation, cell-free supernatants were collected, and IFN-γ production was measured using a Human IFN-γ ELISA set (BD Biosciences) and BD OptEIA™ Reagent Set B (BD Biosciences) according to the manufacturer’s instructions.

### *In vivo* treatment

Therapeutic effects of mesothelin peptide-specific CTLs were investigated *in vivo* in pancreatic cancer experiments. Six- to 8-week-old NOD/SCID common γ-chain knockout (NSG) female mice (Charles River) were used. In total, 10^7^ SUIT2 cells were subcutaneously implanted in the flanks of NSG mice; these cells expressed HLA-A2402 and mesothelin (Day 0). On days 23 and 37, mice received an intra-venous injection of 5 × 10^6^ mesothelin-specific HLA-A2402 restricted CTLs. The untreated mice were injected with PBS as negative controls. Mice were weighed and tumor size was measured once a week.
